# Breath on the Brink: Navigating Anaesthesia Management in a Case With a Mediastinal Mass

**DOI:** 10.7759/cureus.65676

**Published:** 2024-07-29

**Authors:** Shahbaz Hasnain, Hashika Jani, Raj A Pedgaonkar, Parantap Trivedi, Meha Pathak

**Affiliations:** 1 Department of Anesthesiology, Dr. D. Y. Patil Medical College, Hospital and Research Centre, Dr. D. Y. Patil Vidyapeeth, Pune, IND

**Keywords:** bilateral iatrogenic pneumothorax, mediastinum malignancy, ventilatory management, benign mature cystic teratoma, airway collapse, anterior mediastinum

## Abstract

Mediastinal teratomas are rare, often asymptomatic, but clinically significant neoplasms that can manifest with a spectrum of symptoms, frequently attributed to the compression of surrounding critical anatomical structures. Here, we present the case of a 19-year-old male with respiratory distress and chest pain attributed to a large anterior mediastinal mass, ultimately diagnosed as a benign mature teratoma of the thymus. Radiological imaging revealed a large, partially cystic mass compressing the pulmonary arteries, aortic arch, and left main bronchus. Surgical excision was deemed necessary due to symptomatic presentation. Anaesthesia management during mediastinal mass excision posed significant challenges, with prompt sternotomy due to ventilation difficulties after induction. Immediate decompression of the mass improved ventilation and saturation. Despite postoperative complications, including pulmonary leaks necessitating tracheostomy, the patient achieved significant recovery. Anaesthesia strategy was to prioritise avoiding muscle relaxants and maintaining spontaneous ventilation to mitigate airway collapse and hemodynamic instability during induction. Collaboration between anaesthesia, surgical, and intensive care teams is vital for comprehensive preoperative assessment, intraoperative readiness, and postoperative care. This case underscores the importance of interdisciplinary collaboration and meticulous planning to optimise outcomes in patients undergoing surgery for mediastinal teratomas.

## Introduction

The mediastinum, located within the thoracic cavity, is enclosed by the pleura on both sides and extends from the thoracic inlet superiorly to the diaphragm inferiorly. It is anteriorly defined by the sternum and posteriorly by the thoracic vertebrae and is divided into three compartments: anterior, middle, and posterior. The mediastinal space encompasses various structures such as the thymus, heart, major blood vessels, lymph nodes, nerves, and segments of the oesophagus and trachea. Approximately half of the mediastinal masses are situated in the anterior compartment. They mainly consist of thymoma, teratoma, thyroid goitre, and lymphoma [1]. Teratoma is the second most common mediastinal neoplasm. It is a tumour characterised by the presence of tissue or organ elements that resemble the normal derivatives of multiple germ layers [2]. These masses can produce symptoms that are either localised, like cough, or attributed to an obstruction, like chest pain, bulkiness, dyspnoea, dysphagia, facial oedema, and superior vena cava syndrome [1]. Timely surgical intervention is key for treatment in symptomatic cases during which induction of general anaesthesia can be challenging in itself due to physiological changes of the respiratory system like reduction in functional residual capacity, closing capacity, and increased airway pressures worsened by the mediastinal mass.

## Case presentation

**Figure 1:**
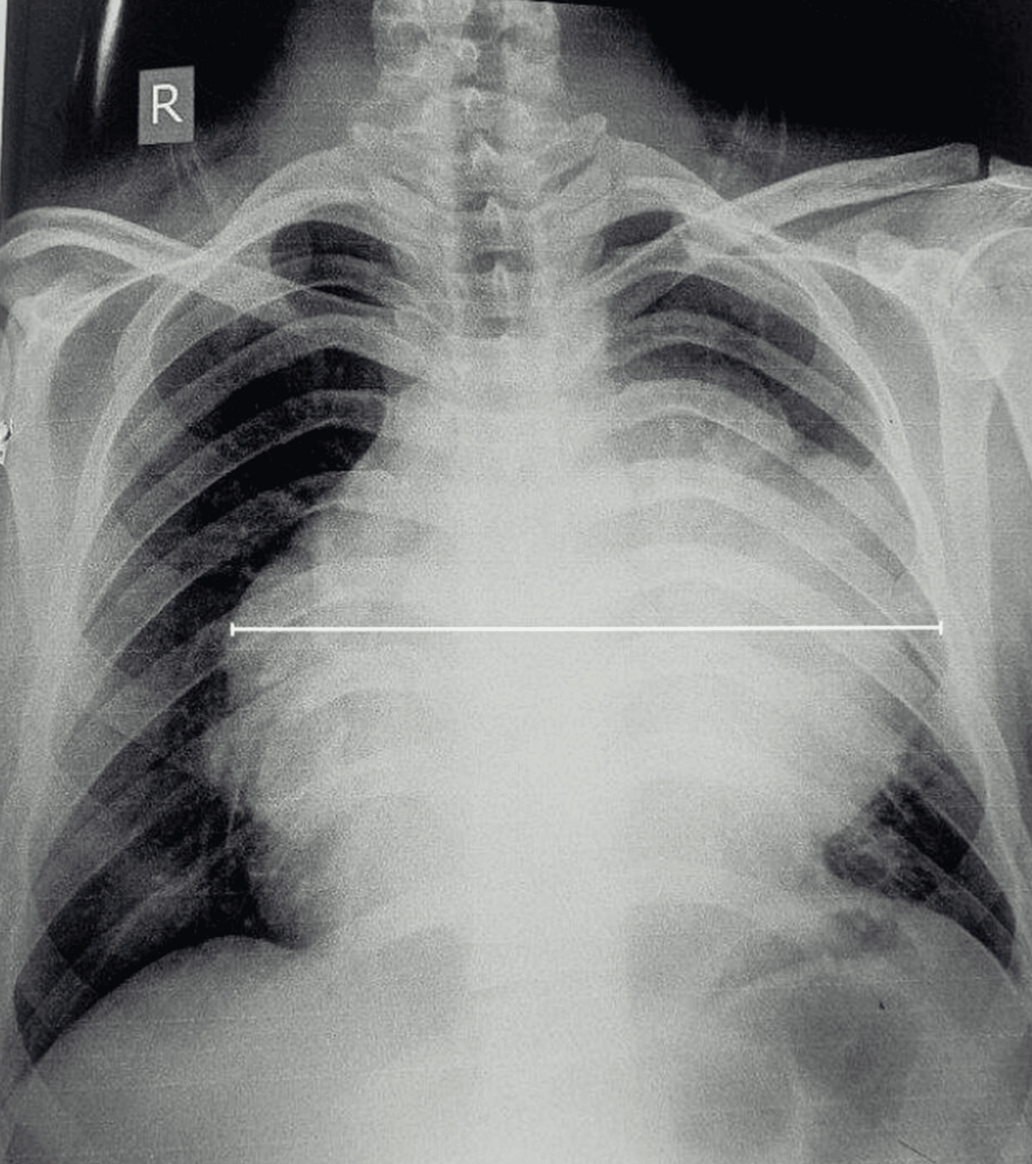
Preoperative chest X-ray showing large homogenous opacity in the mediastinum SICU: surgical intensive care unit

We report the case of a 19-year-old male who presented with breathlessness on exertion and upon lying down, which was relieved on sitting upright; it was associated with chest pain for 15 days. He also had low-grade fever along with dry cough, loss of appetite, and dysphagia for 15 days. His room air saturation was 96% while sitting and 91% while in a supine position. Chest auscultation indicated reduced air entry in the left infraclavicular and midaxillary areas. Chest X-ray showed a widening of the mediastinum with a large homogeneous opacity (Figure 1).

**Figure 2:**
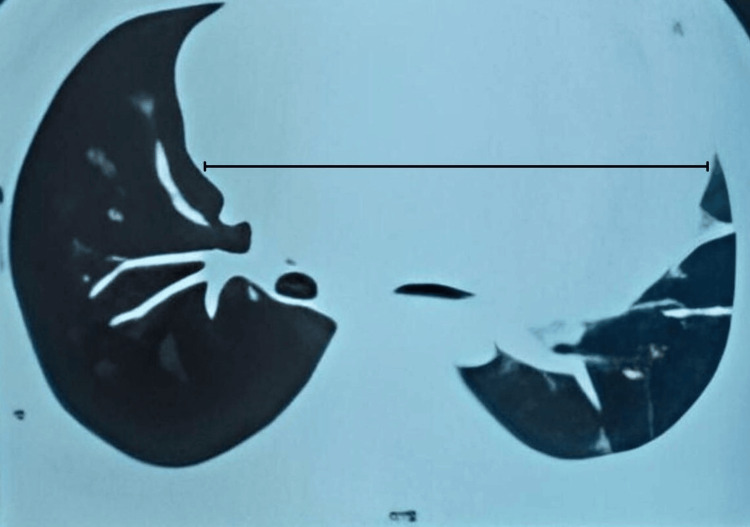
CT scan of the axial section of the thorax showing the mediastinal mass

A CT scan of the thorax revealed a large well-defined anterior and middle mediastinal mass, partially cystic, measuring 18.7 x 10.7 x 12.8 cm (Figure 2). The mass was abutting and compressing the main pulmonary arteries, anterior chest wall, aortic arch, and ascending aorta, and it displaced the left main bronchus. Mild pleural effusion was noted on the left and minimal on the right side. There was also mild pericardial effusion with a maximum thickness measuring 12 mm.

Routine haematological examination was within normal limits. A tissue biopsy of the mass revealed unremarkable fibrocollagenous tissue with thymic elements. An aspirate from the cystic component of the mass showed a haemorrhagic acellular smear.

Mediastinal mass excision was planned under general anaesthesia after a thorough pre-operative assessment and multi-disciplinary collaboration. The patient’s airway was prepared for an awake intubation and then transferred to the operating room. He was placed in a semi-recumbent position with 30° head end elevation. Apart from the routine American Society of Anesthesiologists standard monitoring, the left radial artery was cannulated for invasive arterial pressure monitoring and the right internal jugular vein catheter was secured under local anaesthesia.

After three minutes of preoxygenation with 100% oxygen, the patient was sedated with an injection of midazolam 0.02 mg/kg intravenous (IV) and an injection of fentanyl 1 mcg/kg IV. Meanwhile, the surgical field was prepared for emergency sternotomy. Spontaneous ventilation was maintained, and awake endotracheal intubation was facilitated with video laryngoscopy and bougie. After securing the airway and confirming adequate manual ventilation, general anaesthesia was induced with injection propofol 2 mg/kg IV and inhalation anaesthetics. Only after the surgical team was ready for immediate sternotomy, the patient was shifted to a supine position and an injection of rocuronium 0.6 mg/kg IV was administered.

**Figure 3:**
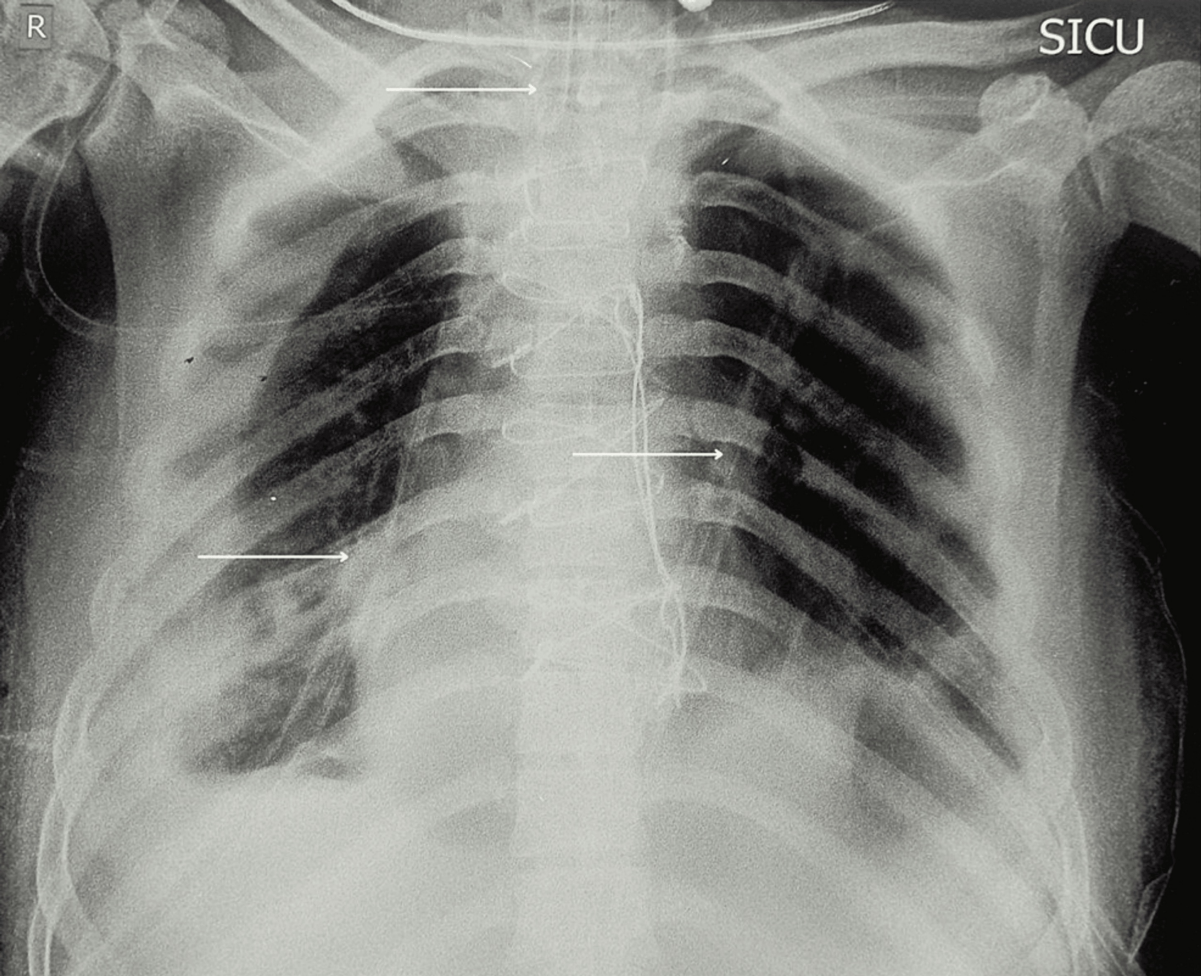
Postoperative chest X-ray showing bilateral pneumothorax and multiple intercostal drains (white arrows) SICU: surgical intensive care unit

Soon after, we encountered severe resistance to manual bag ventilation, resulting in a fall in saturation to 85%, a rise in central venous pressure to 21, peak airway pressure of 42, and end-tidal CO_2_ of 57. The lungs could not be ventilated effectively. Arterial blood gas revealed a pH of 7.24, partial pressure of carbon dioxide of 72 mmHg, and partial pressure of oxygen of 82 mmHg with a saturation of 84%. Immediate sternotomy and aspiration of 600-mL cystic fluid from the mass led to quick decompression and improvement in ventilation and saturation. The remainder of the surgery proceeded smoothly; the mediastinal mass was removed with the stripping of the anterior pleura and pericardium, which was required due to the adherence of the mass. Bilateral intercostal drains were inserted, and the patient was transferred to the intensive care unit (ICU) while still intubated. He was on controlled ventilation overnight for one day and then switched to assisted ventilation. Despite intraoperative care, postoperative complications emerged in the form of bronchopulmonary fistula due to stripping of the pleura and surgical trauma leading to bilateral pneumothorax. Multiple bilateral intercostal drains were secured, and serial X-rays of the chest were done (Figure 3). He required prolonged intubation and tracheostomy.

He continued to receive antibiotics, mucolytic nebulisation, and rigorous chest and limb physiotherapy throughout his stay in the ICU. A sample of the cystic wall was sent for histopathological examination, revealing a mature teratoma of the thymus. After enduring an extended ICU admission and surgical complexities, the patient exhibited remarkable recovery and was discharged six weeks after surgery.

## Discussion

Mediastinal teratomas are extensively documented in medical literature. They typically do not show symptoms; however, in this case, respiratory symptoms were noticed due to the compression of pulmonary arteries and displacement of the left bronchus. Although teratomas are benign masses, surgical excision is indicated when they become symptomatic because the tumour can gradually enlarge, infiltrate neighbouring tissues, and potentially become malignant. Complications such as infection and rupture may also arise [3].

When administering anaesthesia to patients with mediastinal masses, it is crucial to consider potential complications such as airway collapse and hemodynamic instability. Therefore, it is strongly advised to avoid using muscle relaxants and prioritize the maintenance of spontaneous ventilation [4]. Due to the ever-present possibility of untoward events, anaesthesiologists are trained to formulate contingency strategies in the face of potential adversities. As in our case, we refrained from altering the patient's position until the surgical team was fully prepared to proceed with sternotomy promptly. The surgery was planned in collaboration with cardiothoracic surgeons. A rigid bronchoscope, cardiopulmonary bypass, and extracorporeal membrane oxygenation were on standby should the need arise [5,6]. Close monitoring throughout the procedure is also paramount to promptly address any complications.

Extensive surgical dissection can lead to trauma to vital organs, as in our patient, who developed bronchopulmonary fistula, which resulted in bilateral pneumothorax, prolonged intubation, and subsequent tracheostomy. Collaborative efforts between surgical, anaesthesiology, and ICU teams towards preoperative counselling, intraoperative readiness, and postoperative care are imperative for achieving favourable surgical outcomes.

## Conclusions

Providing anaesthesia for patients with anterior mediastinal masses presents a formidable challenge for anaesthesiologists. Accurately identifying individuals at elevated risk of airway obstruction and cardiovascular decompensation remains a significant hurdle. Rigorous preoperative patient evaluation, effective communication with surgical teams, meticulous planning, and readiness to address complications arising from the compression of critical airways and blood vessels is crucial for attaining favourable results of surgery.
